# Point of Care Transesophageal Echocardiogram-guided CPR: Area of maximal compression

**DOI:** 10.24908/pocusj.v10i01.18111

**Published:** 2025-04-15

**Authors:** Yannis Amador

**Affiliations:** 1 Department of Anesthesiology and Perioperative Medicine, Kingston Health Science Center, Kingston, ON, CAN

**Keywords:** Transesophageal echocardiography, cardiac arrest, cardiopulmonary resuscitation, cardiac anesthesiology

## Abstract

During cardiopulmonary resuscitation (CPR), chest compressions are critical for augmenting cardiac output, primarily through left ventricular (LV) compression. However, achieving optimal compression without direct visualization can be challenging. Point of care transesophageal echocardiography (TEE) serves as an invaluable tool for real-time guidance, ensuring accurate chest compression positioning over the LV apex. In this case, initial compressions were misaligned over the left ventricular outflow tract (LVOT) and aortic valve (AV). TEE assessment enabled real-time identification and precise repositioning of compressions to the LV apex, resulting in marked improvements in arterial pressure waveforms and end-tidal CO_2_ (ETCO_2_)—both reliable indicators of CPR quality. This case highlights the critical role of TEE in cardiac arrest management, offering real-time diagnostic insights and optimizing compression mechanics. Integrating TEE into resuscitation protocols enhances the quality of chest compressions, supports hemodynamic stability, and may ultimately improve patient outcomes.

## Background

Both transthoracic echocardiography (TTE) and transesophageal echocardiography (TEE) are indispensable tools for cardiac imaging, each offering unique advantages in clinical practice. However, point of care TEE demonstrates clear superiority over TTE in specific critical situations, particularly during cardiopulmonary resuscitation (CPR) [1]. TTE becomes impractical in this setting due to challenges in acquiring high-quality images and the limited time available during pauses in chest compressions to perform a comprehensive cardiac assessment [2]. Additionally, TTE imaging is often compromised by anatomical barriers such as the lungs, ribs, and suboptimal acoustic windows [1]. In contrast, TEE overcomes these limitations through its esophageal positioning, which bypasses thoracic anatomical barriers and provides uninterrupted, superior visualization of cardiac structures. This makes TEE a highly effective modality, particularly in emergent and peri-arrest scenarios [1,3].

TEE is invaluable for identifying critical cardiac conditions that inform clinical decision-making during shock and cardiac arrest. These include pericardial effusion or tamponade, global or regional ventricular dysfunction (right or left ventricular (LV) failure), major regional wall motion abnormalities, cardiac standstill, ventricular fibrillation, and systolic anterior motion (SAM) of the mitral valve [1]. The superior imaging capability of TEE in these time-sensitive conditions enhances diagnostic accuracy, facilitates targeted interventions, and ultimately improves patient outcomes during resuscitation efforts [1].

## Case

**Figure 1.**
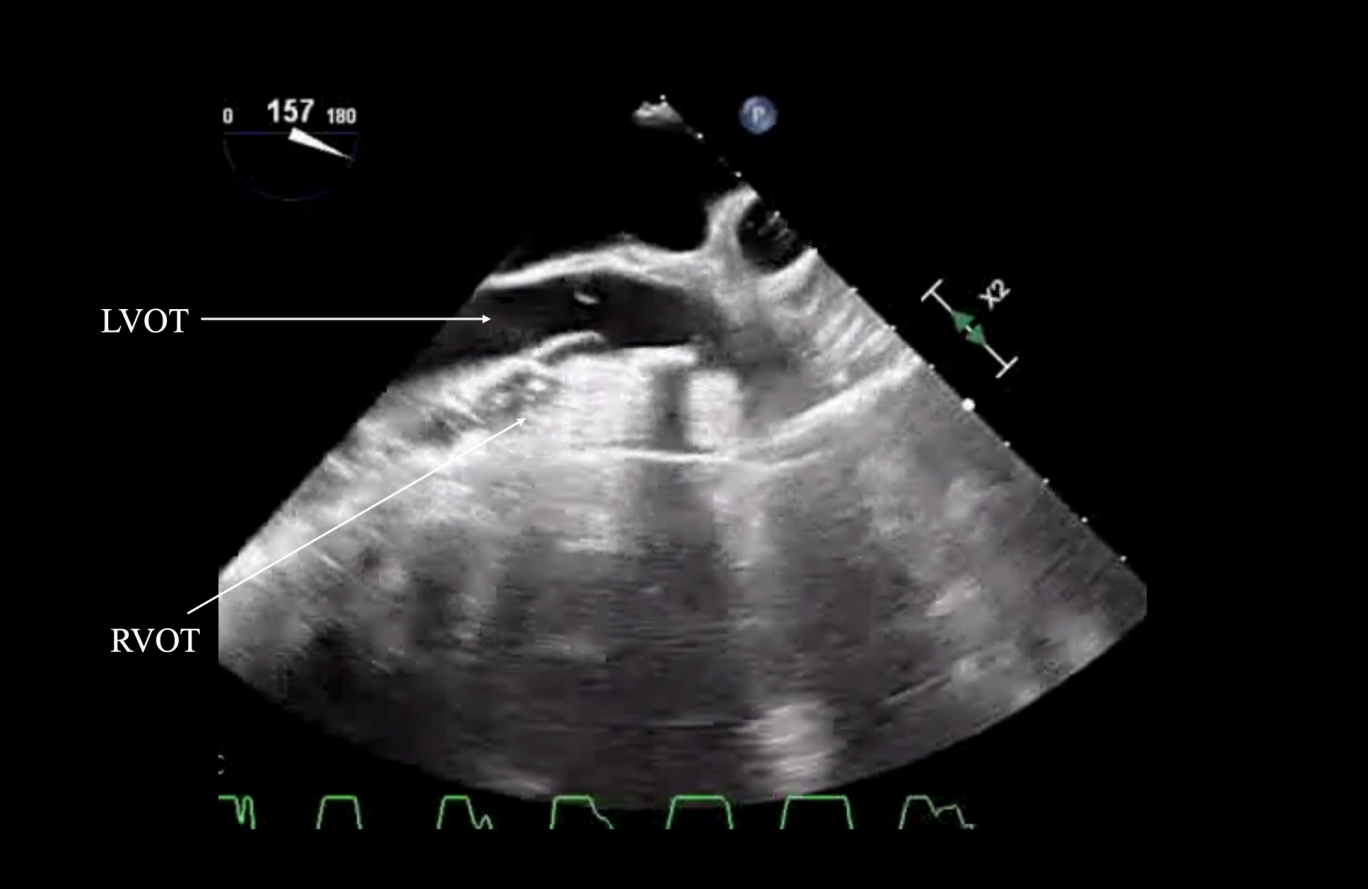
Mid-esophageal aortic valve long-axis (ME AV LAX) view depicting a compression of the right ventricular outflow tract (RVOT). Just before the aortic valve (AV), the left ventricular outflow tract (LVOT) and mid-esophageal aortic valve short axis (ME AV SAX) view shows the AV compression. The RVOT, LVOT and AV compressions occur during chest compressions. Further visual information in Video 1.

A 77-year-old man scheduled for coronary artery bypass grafting (CABG) experienced cardiac arrest immediately following the induction of anesthesia. His medical history was significant for hypertension, type II diabetes mellitus, and triple-vessel coronary artery disease with a proximal left main occlusion. The accompanying imaging demonstrates the role of point of care TEE in optimizing CPR during cardiac arrest. Initial chest compressions were inadvertently focused over the left ventricular outflow tract (LVOT) and aortic valve (AV), as shown in Figure 1 and Video 1. This misalignment resulted in partial obstruction, potentially compromising the hemodynamic efficacy of CPR.

Under TEE guidance, the site of chest compressions was adjusted to the LV apex, relieving the obstruction of the LVOT and AV (Video 2). This adjustment highlights the critical role of TEE in ensuring precise compression targeting. Maximizing LV compression can improve resuscitation mechanics and possibly the outcomes. While it is impossible to definitively determine the extent to which the correction of chest compression positioning influenced the outcome in this case, it is strongly believed that the intervention played a critical role. Ultimately, the improvements observed in arterial waveforms and ETCO_2_ levels following TEE-guided adjustments reinforce its utility as a quality control tool in CPR management. This case adds to the growing evidence supporting the integration of TEE into advanced cardiac life support (ACLS) protocols, particularly in perioperative and critical care settings.

## TEE-guided hands correction

During the resuscitation attempt-ongoing CPR, the TEE revealed that compressions were not effectively targeting the LV apex. Upon this observation, the surgical team was instructed to adjust hand placement towards the apex. This immediate correction resulted in improved compression efficacy, as evidenced by enhanced LV “squeeze” on TEE imaging. Additionally, the LVOT/right ventricular outflow tract (RVOT) showed no collapse.

## Discussion

Point of care TEE has emerged as an indispensable diagnostic tool during cardiac arrest, providing real-time imaging that allows clinicians to confirm or exclude life-threatening conditions. Beyond diagnosis, TEE significantly enhances the quality of CPR by guiding and optimizing chest compression positioning, which is often suboptimal when performed blindly[1,3].

While definitive clinical trials confirming the universal efficacy of TEE in cardiac arrest are lacking, a growing body of evidence strongly supports its benefits in life-threatening scenarios. TEE facilitates more targeted interventions and improves the precision of resuscitative efforts, underscoring its critical role in modern cardiac arrest management [1,4,5]. This case highlights the pivotal role of TEE in real-time guidance of CPR during cardiac arrest. By enabling direct visualization of cardiac structures, TEE facilitated prompt adjustments to CPR technique, ensuring accurate compression positioning and optimal hemodynamic performance. Incorporating TEE into resuscitation protocols can enhance the likelihood of favorable patient outcomes, particularly in scenarios where traditional approaches may be suboptimal.

In both medical and post-surgical settings, TEE offers substantial advantages for the diagnosis and management of cardiac arrest. By facilitating the exclusion and treatment of critical conditions, TEE supports targeted interventions tailored to specific pathologies. For example, conditions such as pericardial tamponade and regional wall motion abnormalities require markedly different treatment strategies, which can be rapidly identified using TEE [1]. Furthermore, during cardiac arrest, specific interventions may inadvertently exacerbate pathology if not carefully monitored. For instance, the administration of epinephrine into an empty left ventricle can precipitate SAM of the mitral valve, which compromises cardiac output. TEE allows for immediate recognition of such complications, enabling prompt intervention to optimize resuscitation efforts [5]. These examples highlight the crucial role of TEE in guiding and managing rescue efforts, particularly in complex clinical scenarios. The ability to visualize cardiac function and hemodynamic status in real time emphasizes the importance of TEE as a core component of ACLS [6,7].

TEE plays a critical role in diagnosing, guiding, and optimizing CPR during cardiac arrest. By enabling precise visualization, TEE improves the accuracy of chest compression placement and facilitates the identification or exclusion of treatable pathologies, ensuring targeted interventions in complex medical scenarios. This enhanced precision not only optimizes resuscitation mechanics but also significantly improves patient outcomes [4,7]. The expanding applications of TEE in critical care underscore the need for clinicians to master its use and remain up-to-date with evolving techniques. As an indispensable tool in cardiac arrest and critical care management, TEE empowers physicians to make real-time, informed decisions. These ultimately contribute to more effective patient care and successful resuscitation outcomes.




